# A hybrid deep learning model for user story effort estimation

**DOI:** 10.1371/journal.pone.0353348

**Published:** 2026-07-17

**Authors:** Saadia Malik, Muhammad Hamid, Muhammad Saleem

**Affiliations:** 1 Department of Information Systems, Faculty of Computing and Information Technology, King Abdulaziz University, Rabigh, Saudi Arabia; 2 Department of Computer Science, Government College Women University, Sialkot, Pakistan; 3 Department of Industrial Engineering, Faculty of Engineering, King Abdulaziz University, Rabigh, Saudi Arabia; University of Kerbala, IRAQ

## Abstract

Accurate effort estimation of user stories is a key challenge in agile software development due to both subjectivity and the complexity of natural language requirements. This paper proposes a hybrid Deep Learning (DL) model for data driven effort estimation using large scale textual data and advanced semantic modeling. One of the significant contributions is the development of a dataset of 6,956 user stories which was collected from many heterogeneous sources and then meticulously cleaned and refined to 4,079 high quality instances, by using a systematic preprocessing and expert validation. Following a Design Science Research (DSR) methodology, a hybrid model integrating a pre-trained Bidirectional Encoder Representations from Transformers (BERT) encoder with a Long Short-Term Memory (LSTM) is developed to capture both contextual semantics and sequential dependencies in user story description. The DL model is evaluated against multiple Machine Learning (ML) baselines using a robust multi-metric framework. Experimental findings show the superior performance of the proposed model with a Mean Absolute Error (MAE) = 0.6481, Root Mean Square Error (RMSE) = 1.4559 and  = 0.6581, which is a huge improvement over the conventional methods. To ensure practical relevance in discrete Scrum planning, the continuous model outputs were mapped to the standard Fibonacci sequence, achieving a classification accuracy of 72%. To guarantee the consistency of performance improvements, the statistical validation is done with the Wilcoxon Signed-Rank Test to show that the improvements are significant (p < 0.05). Moreover, the model is operationalized as web based decision support system to enable real time estimation in agile software development. The results reveal the efficacy of using large scale curated datasets alongside hybrid DL model to reduce estimation bias and boost predictive quality to deliver a scalable and strong solution for intelligent agile project management. Future work will focus on integrating eXplainable AI (XAI) techniques and validating the model across real world industrial datasets to further enhance transparency and generalizability.

## 1. Introduction

The modern software industry is characterized by an ongoing tension between the demand for rapid development cycles and the necessity of maintaining high quality standards. To address these competing priorities, organizations have progressively shifted from rigid, traditional development models to more flexible and iterative agile methodologies. Within this paradigm, scrum has emerged as the most widely adopted methodology over the past two decades, demonstrating significant dominance in both industry adoption and practical effectiveness compared to alternative approaches [1]. Scrum helps in developing the projects by breaking down the complex projects into manageable iteration called sprints over fixed period of time. During these sprints, teams are focused on giving certain functional requirements that are often written down for them in the form of user stories. However, although the implementation of this iterative model is very flexible, at the same time it poses a fundamental and constant operational challenge, accurate estimation of the effort is required to implement each user story [2]. This estimation process is a critical problem, as inaccurate estimations leads to failure of project, resulting in misallocation of resources, budget overruns and missed deadlines [3]. The difficulty is compounded by a range of context dependent factors known as “effort drivers” such as task complexity and clarity of requirements. To overcome this, teams traditionally use a subjective approach such as Planning Poker. However, research has shown that these methods tend to be time-consuming, and highly susceptible to human bias, which can delay the entire planning process [4]. Consequently, the focus has moved into data-driven ways, and researchers now increasingly explore ML models to offer objective predictions [5,6].

Artificial Intelligence (AI) provides an effective solution to overcome the shortcomings of manual estimation where ML and DL are becoming common approaches. However, one of the significant research gaps is that traditional ML models work well with numerical data, but are less successful with interpreting the most critical information in Scrum, the natural language text of the user stories. This is the particular challenge that DL solves. As confirmed in recent extensive surveys, DL is being used successfully in various software engineering tasks such as effort estimation because of its sophisticated characteristics of natural language processing (NLP) [7]. DL models capture semantic representations of textual requirements through contextual embeddings, within a user story to automatically discover the patterns of complexity leading to much more objective and accurate effort prediction.

In order to solve these challenges, this study aims to answer the following Research Question:

*To what extent can a hybrid DL model, trained with a large-scale, expert-curated dataset, address the issues of subjectivity and inaccuracy in user story effort estimation?*

To provide answers to this research question, this paper makes the following main contributions:

1) By incorporating data from several platforms and repositories a comprehensive dataset of 6,956 user stories is developed. To guarantee reliability and consistency dataset undergo preprocessing, standardization and expert driven validation by industrial Scrum practitioners. Following data preprocessing, a high quality dataset of 4,079 user stories is kept for model training and evaluation.
2) A novel DL model is developed which combines BERT based contextual embeddings with an LSTM network to estimate effort of user stories. This model uses BERT’s ability to represent semantics and LSTM’s ability to model long range sequential dependencies to improve the feature extraction and prediction accuracy.
3) An experimental setup is used to compare the proposed model’s performance to established baselines. The Wilcoxon Signed Rank Test is used to statistically validate the superiority and robustness of the proposed model. In addition, a classification-oriented evaluation strategy is introduced by mapping continuous model predictions to standard Agile Fibonacci story point categories {1,2,3,5,8,13,20}. The effectiveness of this mapping is further evaluated using accuracy, precision, recall, F1-score, and confusion matrix analysis to improve the practical applicability of the model in Scrum sprint planning.
4) The proposed model is materialized as a web-based decision support system to close the gap between research and practical application. This system enables agile practitioners use the BERT-LSTM model to estimate effort in real time by reducing human bias and makes Scrum-based project planning more data-driven and reliable.

The remainder of this paper is organized as follows: Section 2 is a detailed review of the relevant literature. Section 3 explains the research methodology from data collection to the design of the proposed DL model. Section 4 shows the experimental results, and finally, section 5 concludes the study and shows the future work.

## 2. Literature review

Effort estimation has always been one of the basic challenges in agile software development. Despite the fact that Scrum has been implemented widely throughout the world, due to the subjectivity of humans and the complexity of requirement the accuracy of the estimating user story effort is affected. While the existing developmental process uses traditional expert-based techniques such as Planning Poker [8], these methods are naturally prone to human bias and inexperience team [2]. Empirical evaluations, like the one by Pozenel et al. [4], show that despite being standard, manual methods are seriously inefficient in comparison to automated systems. However, one recurrent drawback of these empirical assessments is that they tend to rely on small-scale dataset, due to a lack of understanding of the dynamic data volatility of industrial undertakings. More critically, these studies tend to report results without strong statistical validation so the comparative advantages remain scientifically unproven.

The disadvantages of the manual ways shift the paradigm to data driven ML methods. Studies by Mahmood et al. [9] and Alsaadi et al. [10] have proved that ensemble techniques could work better than singles. Nevertheless, these studies are burdened with a critical data-scarcity issue; for example is the use of a dataset of only 140 user stories that impacts the model ability to learn complex pattern of the user stories [10]. Furthermore, a systematic review by Hariyanti et al. [11] finds that the modern effort estimation literature is heavily biased towards structured or semi-structured data. Successful models proposed by Lavazza et al. [12] and Arora et al. [13] have a very high precision but are still limited because they are dependent on pre-formatted inputs such as Function Points. By avoiding the raw and unstructured narrative form of the text-based user stories, these methodologies ignore the source of the complexity in requirements. Additionally, these studies usually assess a single proposed model without having a fair head-to-head experimental comparison of the model against a diverse set of modern ML baselines on the same data. Another major gap in research for non- DL is reliance on manual pre-categorized inputs. For example, the Bayesian Network approach by Turic et al. [14], Intelligent recommender system by Hamid et al. [15] and the ELM model by Kumar et al. [16] resulted in high accuracy but were strictly based on project metadata or expert-defined complexity labels. This dependency essentially puts the burden of estimation back on human intervention and is not adequately providing an automated solution to estimation. Furthermore, a general weakness among this generation of studies is the absence of strong statistical significance testing. Results are usually given in terms of simple point estimates (e.g., MAE), without paired significance analysis (e.g., Wilcoxon test), in order to demonstrate that performance improvement is not due to random data partitioning. Furthermore, recent work by Rasheed et al. [17] introduced an ensemble of Ridge Regression, Extra Tree and MLP achieving an MAE of 0.68.

Moreover, DL has recently become a state-of-the-art solution for tasks that demand semantic understanding [7]. Yet, even in the DL point of view, there is a cardinal ‘quality vs. scale’ divide. The replication study conducted by Tawosi et al. [18] on 31,000 stories found that high error rates occur with advanced models such as Deep-SE, if they are intended to be trained on uncurated and noisy data. This represents a two-fold research gap in the state-of-the-art: (i) a severe lack of high quality expert normalised data sets, and (ii) a lack of comprehensive benchmarking in which DL models are strictly compared with traditional ML baselines.

Existing literature provides limited solutions that simultaneously address semantic modeling, dataset quality, and statistical validation. The present study directly addresses these multi-faceted limitations by creating a new expert-curated dataset as well as deploying a hybrid BERT-LSTM model subjected to both multi-metric benchmarking and paired statistical significance testing to set a new, robust performance benchmark from automated agile effort estimation.

## 3. Proposed Methodology

**Fig 1:**
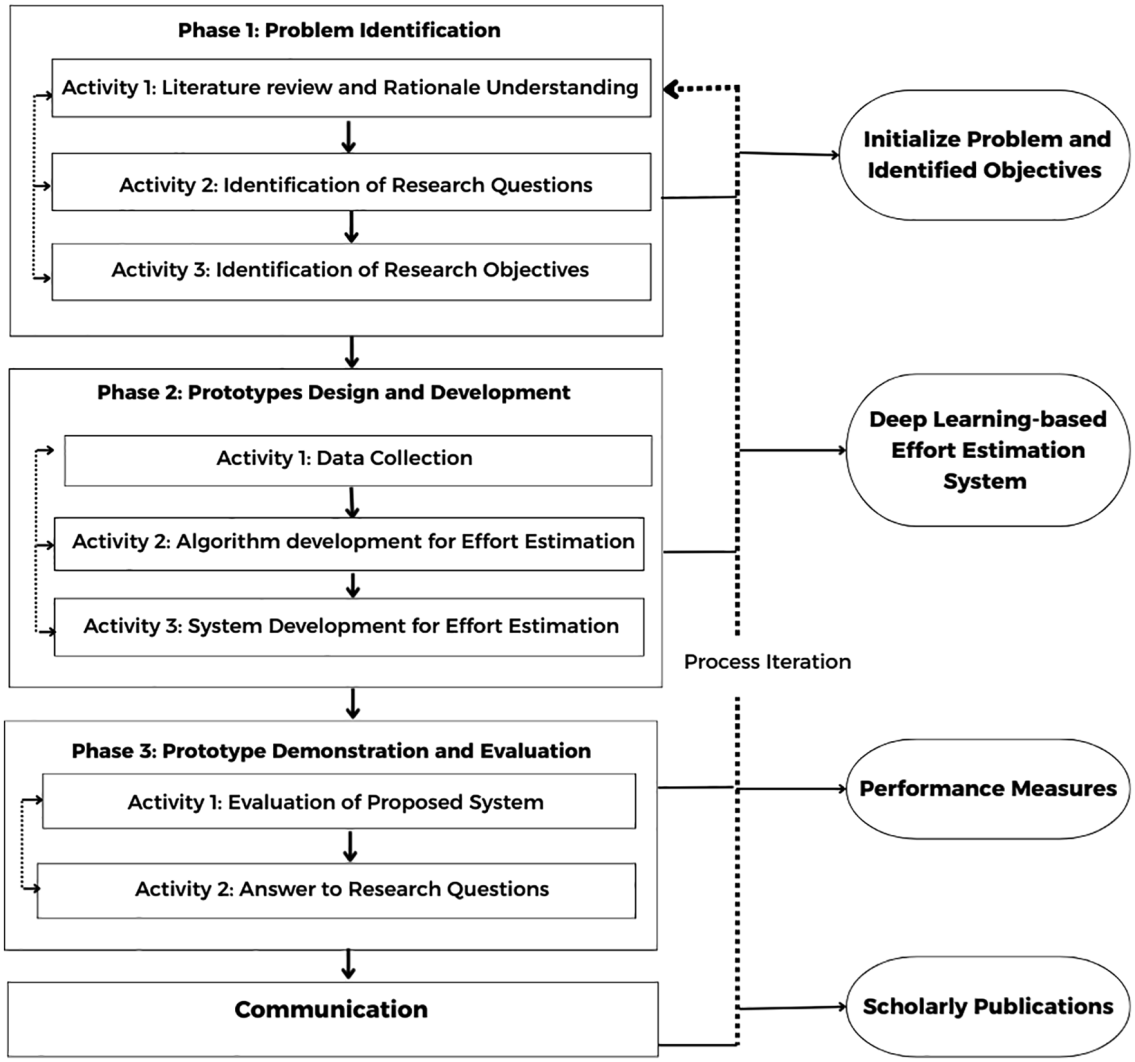
Workflow of DSR Methodology.

This research follows the DSR methodology to fill the gap identified in literature review. DSR is a practical and problem-solving approach that is focused on developing and testing innovative artifacts. The process is divided into three key phases namely: Problem identification, Prototype design and development and finally, Prototype demonstration and evaluation as shown in Fig 1.

a) The first phase was identification of the problem and motivation to investigate and define the research problem. This phase involved three main activities: literature review in order to identify the existing gaps in research for AI based effort estimation; identifying specific research questions to outline the core problems; setting measurable goals for an effective solution.
b) The second phase, prototype design and development included three main activities: thousands of user stories were extracted from several platforms and then processed to become a unified, refined and standardized data set. Next, a hybrid DL model was created, designed to specifically understand the deep meaning in the text of the user story to accurately predict the story points. Finally, this algorithm was implemented as a simple web based decision support system which scrum master can easily use.
c) The third phase, prototype demonstration and evaluation, consisted of two further activities: evaluation of the proposed model with the goal of rigorously testing its accuracy and reliability using standard performance metrics and providing an answer to the research questions by validating whether the artifact fulfils the objectives defined by Phase 1, thus confirming its contribution to efforts estimation improvement. The detail of each phase is below.

### 3.1. Problem Identification Phase

The first phase of this research identified the fundamental challenges in accurate estimation of the effort of user stories in Scrum. A review of the existing literature, presented in Section 2, revealed two main gaps that this study seeks to resolve. First, traditional and simpler ML models are unable to interpret the rich, unstructured nature of text of user stories, restricting their accuracy. Second, progress in this field is seriously hindered by lack of large, good quality, and consistent datasets, which is the basis for training DL models. This gap of needing both a model and dataset provided a clear direction for this research that involve compiling and standardizing a large-scale dataset and state-of-the-art BERT-LSTMs model to utilize it.

### 3.2. Prototype Design and Development

This phase had three main activities: First, thousands of raw user stories were gathered from different independent platforms and repositories and then aggregated into a single and refined dataset. Next a novel hybrid DL model was created which was specifically architected to semantically interpret the deep contextual meaning within text of user stories to accurately predict story points. Finally, this algorithm was incorporated in a web-based decision support system to make it a useful and accessible tool to agile practitioners.

#### 3.2.1. Data Collection.

The development of reliable efforts estimation models for agile software development is frequently hampered by the lack of publicly available datasets with both structured user stories and accurate story points. Industrial project data are often proprietary, and are not readily available for sharing because of the confidentiality restrictions, limiting the availability of large-scale training data for ML and DL models. To overcome such limitation, a complete dataset was compiled based on a systematic process from multiple publicly available sources and expert data validation.

The compilation process of the data set include three main phases: (i) gathering of raw project artifacts from various repositories, (ii) structural standardization of requirements into the format of a user story and (iii) effort estimation with expert support. The final user story dataset that has been curated consists of 6956 stories from the user with validated story point estimates.

**a) Data Sources**

Three primary sources were used to gather user stories because they guaranteed a variety of data and enough volume.

1. Public Agile Dataset

The first dataset was obtained from a publicly available agile project dataset originally developed for academic research purposes. In this dataset, there were 160 user stories and several project related attributes, such as task complexity, no of task, type of development, database involvement, and the involvement of team members. However, there were also predefined story point estimates in the dataset. Nonetheless, as the aim of this study is to estimate effort of user story using natural language processing, only user story descriptions and story point values were saved. The other auxiliary attributes were eliminated in the data cleaning stage. This dataset was well structured and included user stories with valid effort labels, but its small size constrained its utility in training the DL models [19,20].

1. GitHub User Stories

To increase dataset size, additional user stories were collected from 27 open source software projects from public GitHub repositories [21]. Based on these projects, 1,680 user stories were derived. These stories usually adhered to the standard agile format and had functional descriptions. An important limitation of this dataset was that story point estimates were not available and are needed as ground truth labels for supervised effort estimation models.

1. JIRA Issue Repository

The third data source was 5,116 issue reports derived from JIRA issue repositories [22], which are publicly available and have also been used for previous empirical study in the area of effort estimation [23]. Unlike GitHub stories, these issues already had story point annotations. However most JIRA entries were written as a short technical task or developer note and not a structured user story. For example, many entries were written in the format implement login validation or fix API authentication bug that don’t include the narrative structure usually used in agile user stories. This inconsistency decreases the ability of natural language processing models in understanding semantic complexity patterns. Therefore such issues needed to be reformulated into standardized user story description.

**b) Data Standardization and Expert Validation**

To ensure consistency and reliability to the dataset, the data curation process was carried out with expertise of Scrum Masters and senior software developers of an industry partner organization. The experts oversaw some of the critical steps in the dataset preparation process.

**1. Cleaning and Attribute Selection:** For the academic dataset which contained 160 user stories, text of the user stories and corresponding story point estimates were retained. All additional attributes such as task counts, type of development, presence of databases and team information were removed in order to keep consistency with the textual datasets gathered from other sources.
**2. Estimation of GitHub Stories:** Since 1,680 GitHub user stories did not have story point annotations, the expert panel went through structured estimation sessions. The experts evaluated each story on the basis of functional complexity, implementation effort, technical dependencies, and development scope. Story Points were determined with expert consensus to ensure reliable effort estimation.
**3. Reformulation of JIRA Issues:** The 5116 JIRA issues were manually reformulated into the canonical agile user story format: “As a <role>, I want <feature>, so that <benefit>.” During this process experts made sure to maintain the original functional purpose of each issue even though they were changing the linguistic clarity and the completeness of the narrative.
**4. Cross-Dataset Consistency Verification:** After the reformulation and the estimation phases were finalized, a complete review of all stories across three data sources was conducted to verify the consistency in the estimation scale of the point values. This process was used to eliminate inconsistencies that may arise due to variance in project areas or estimation activities across repositories.
**c) Final Dataset**

After going through the data cleaning, standardization, and expert validation phases, all sources were integrated into one consolidated dataset. The final dataset consists of 6956 user stories with a valid story point estimation for each. Each entry of the dataset is comprised of:

• A standardized agile user story narrative
• A validated story point estimate representing development effort
• Consistent estimation scales across all instances

**Fig 2:**
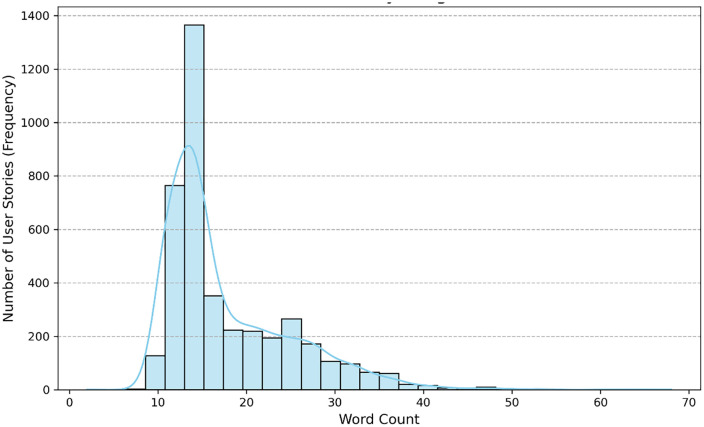
Distribution of user story length in dataset.

**Fig 3:**
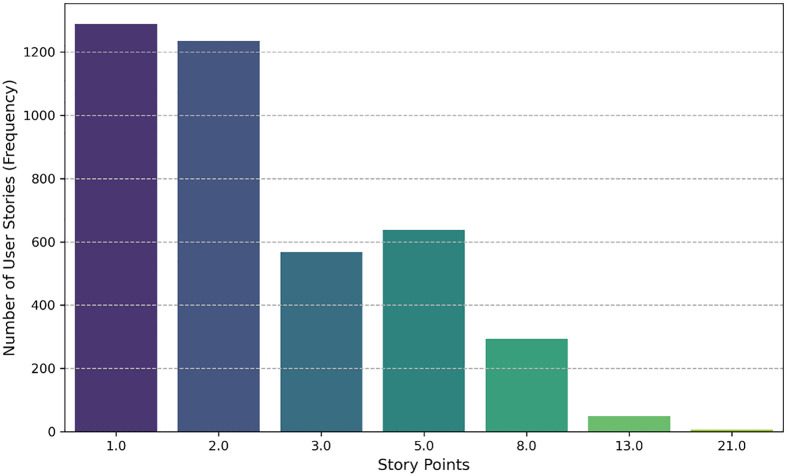
Distribution of story points assigned to user stories in Dataset.

**Fig 4:**
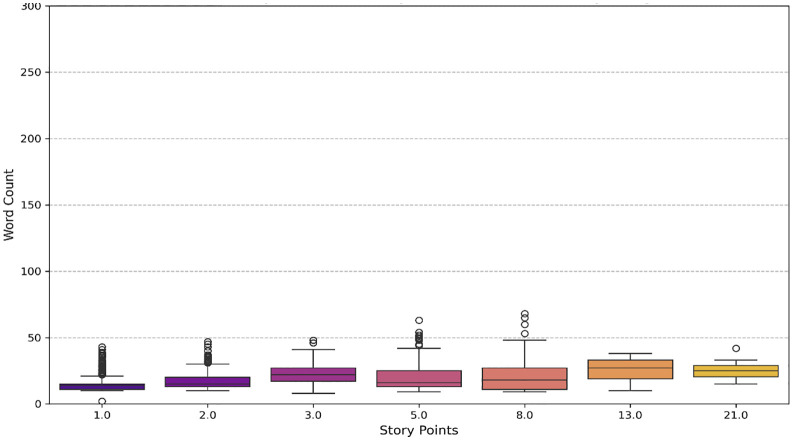
Relationships between user story length and assigned story points.

This curated dataset combines structured natural language requirements with reliable effort labels, making it suitable for training models for agile software effort estimation. Representative examples of user stories and their corresponding story point estimates are presented in Table 1. To provide a comprehensive overview of the curated dataset’s characteristics, Fig 2 illustrates the distribution of user story lengths, indicating the range of narrative complexity captured from the various repositories. Fig 3 presents the distribution of assigned story points, confirming that the dataset maintains a balanced representation across the Fibonacci sequence {1, 2, 3, 5, 8, 13, 20}. Furthermore, Fig 4 visualizes the correlation between user story length and the assigned story points, highlighting how requirement verbosity influences complexity as perceived by the experts. These distributions collectively confirm that the dataset is diverse and spans varying levels of effort, providing a robust empirical basis for the deep learning model.

**Table 1:** Illustrative Examples of User Stories and Assigned Story Points.

ID	User Stories	Expert Estimated story points
US_01	As a registered user, I want to reset my password via email so that I can regain access to my account if I forget it.	2
US_02	As a manager, I want to generate a monthly sales report in PDF format so that I can analyze team performance.	5
US_03	As a developer, I want to integrate the Stripe API for multi-currency payments to support international customers.	8
US_04	As a system administrator, I want to migrate the legacy database to the cloud infrastructure with zero downtime.	13

#### 3.2.2. Algorithm for Effort Estimation.

**Fig 5:**
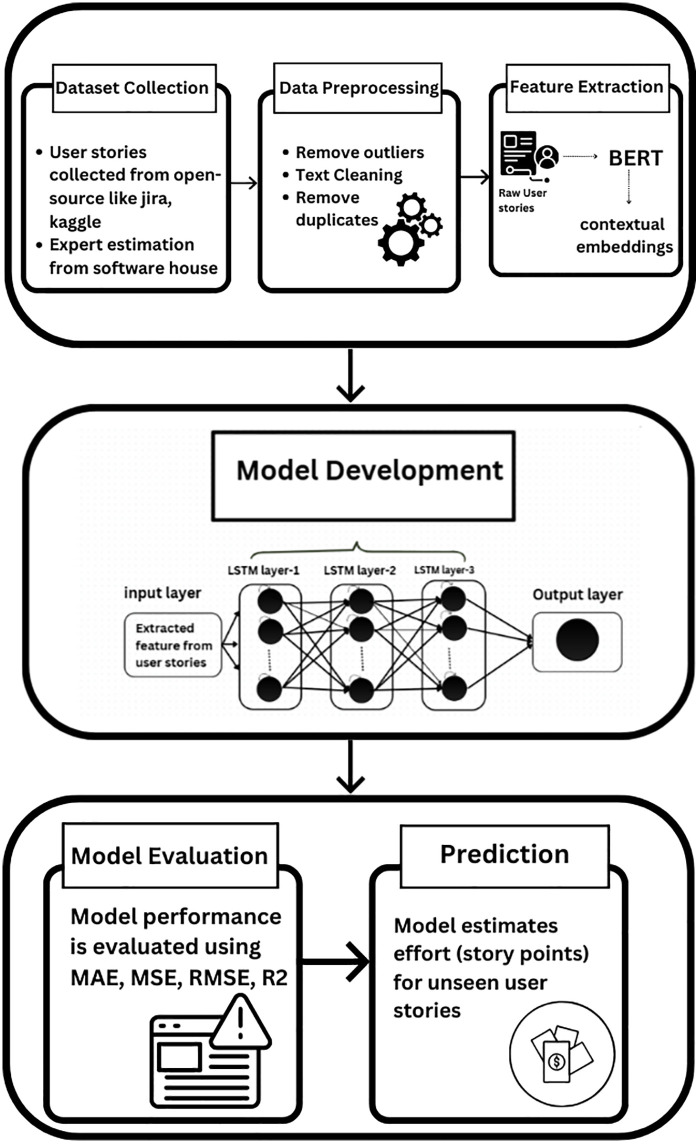
Proposed DL Model for effort estimation.

The proposed algorithm is an end-to-end pipeline, which captures predictive patterns of user story description. With a DL model, the model suggests the semantic context and complexity of the text, rather than binary uses like the use of keywords. Fig 5 illustrates this complete procedure of text input to a final prediction of the story points.

**a) Data Preprocessing**

To ensure the dataset’s overall quality, the initial aggregated collection of 6,956 user stories was meticulously and systematically processed and cleaned. Dataset was collected from several different platforms and a strict standardization pipeline was used. The first step was to read raw CSV files and correct inconsistencies in the file structure. All user story narratives were then converted to lowercase letters and irrelevant special characters and additional whitespaces were excluded to ensure a consistent text structure. The dataset was cleaned to exclude exact and near duplicate user stories, in order to explicitly avoid data redundancy and data leakage. This global deduplication process accurately identified and eliminated 2,088 identical narratives shared across the different repositories with absolute uniqueness of each sample. After this, a critical data integrity check was performed where 502 records were removed because they lacked a narrative or story point label. Furthermore, outliers were addressed by removing user stories with an effort estimation of more than 20 story points. This decision is scientifically justified by agile best practices: requirements with a value greater than 20 points are usually called “Epics” and not atomic user stories. In professional industrial environments such large tasks are mandatory to be broken down into smaller granular stories before sprint planning. Therefore, limiting the scope of the 1–20 Fibonacci range will ensure that the model is limited to granular estimation of effort and maintains a representative distribution of typical sprint tasks. This rich and rigorous curation produced a refined, reliable and structured data set of 4,079 unique user stories, which serves as foundation for training the DL model. The overall preprocessing pipeline and the resulting dataset size at each stage are summarized in Table 2.

**Table 2:** Overview of data preprocessing steps and final dataset composition.

Preprocessing Step	Number of User Stories
Raw collected dataset	6,956
Duplicate stories removed	2,088
Missing values removed	502
Outliers (Story Points > 20) removed	287
Final cleaned dataset	4,079

**b) Feature Representation and Selection**

The methodology of feature representation in this study is based on the implicit power of DL to extract features directly from raw text. Instead of manual feature engineering, the model is architected to learn predictive patterns in the description of user stories through a multi-layered approach in an automatic manner. BERT is used for the purpose of contextual feature representation in which the self-attention mechanism of the model performs a first layer of feature selection based on the importance of certain words depending on their context. Subsequently, LSTM layer are used for sequential feature selection where the long-range dependency and temporal patterns are identified in the requirement description. This enables the model to determine how a particular order of the functional steps affects the overall requirement complexity.

**c) Effort Estimation Model**

The main part of this research is an advanced hybrid DL model combining a fine-tuned encoder of BERT with an LSTM model. This model is created to understand the deep contextual meaning of the user story description and sequential patterns in order to accurately predict effort. While transformer-based architectures like BERT are very good at learning contextual relationships between words using self-attention mechanisms, user story description can include both semantic context and structural patterns that are associated with task descriptions. In this work, BERT is used as a contextual encoder in order to generate rich semantic embeddings of user stories. These embeddings are then passed through an LSTM layer which assists in modeling sequential patterns which are contained in the narrative structure. This hybrid design allows this model to benefit from contextual language representations and also capture sequential information that might affect estimation of efforts. To attain an optimal performance the following hybrid model approach was adopted: BERT as a feature extractor specifically for the domain, resolving the issue of the vocabulary gap, and the LSTM as a condition processor of the presented sequence, taking into consideration the dependencies in the structure.

The architecture is made of a series of consecutive layers:

1. **Input Layer:** Accepts tokenized input text in the form of numerical IDs. Maximum length of the input with 256 tokens.
2. **BERT Encoder:** Uses a pre-trained BERT base-uncased model, trainable flag = True applies it in agile specific narratives, high dimensional embedding and context-aware embeddings
3. **LSTM Layer:** Possess 64 units with dropout, dropout recurrent of 0.3 that will prevent overfitting by processing the sequences. The contextual token embeddings produced by the BERT encoder are fed as sequential inputs to the LSTM layer so that the model can be further used to handle the narrative sequence of the user story.
4. **Intermediate Dense Layer:** It contains 32 number of neurons having a ReLU activation function for non-linear feature mapping.
5. **Final Dense Regressor:** To predict the continuous value of Story Point, a single neuron is used (with linear activation function).

Because the context (what the feature does) and sequential complexity (how it is implemented) are integrated in multiple ways, this ensures the model reproduces exploratory coverage. The last Dense Regressor layer is responsible for mapping these semantic embeddings to a continuous numerical value as an effective way of reframing the task of effort estimation into a regression task. This dual processing capability plays a crucial factor in this experimental results ability to perform such predictive tasks with the observed precision.

**d) Hyperparameter Optimization**

To identify a suitable configuration for the hybrid model, the systematic grid search strategy was adopted in a multi-dimensional search space. The optimization was done to balance between the model capacity and the generalization capacity. The evaluated ranges and the final selected values are shown in Table 3.

**Table 3:** Hyperparameter Grid Search Space and Optimal Values.

Parameter	Search Space (Grid)	Optimal Value
BERT Architecture	Fixed (Pre-trained)	bert-base-uncased
Max Sequence Length	{128, 256, 512}	256
Learning Rate	{2e-5, 3e-5, 5e-5}	2e-5
Batch Size	{8, 16, 32}	8
LSTM Units	{32, 64, 128}	64
Dense Layer Units	{16, 32, 64}	32
Dropout Rate	{0.2, 0.3, 0.4}	0.3
Optimizer	{Adam, AdamW, RMSprop}	Adam
Max Epochs	{5, 10, 15}	10

The choice of these values was made on the basis of a rigorous empirical evidence. Specifically, the hybrid configuration utilizes 64 LSTM units to capture temporal dependencies, followed by a dense layer of 32 units to ensure sufficient capacity without the problems of over-parameterization. A dropout rate of 0.3 was found as the optimum value for regularization in order not to overfit while still maintaining the learning efficiency. Furthermore, training was limited to 10 epochs, as the model was consistently getting stable convergence and very low validation loss before reaching such a threshold, which is typically caused by the use of the early stopping mechanism.

**e) Pseudo code of model**

**Input:** User Story Text

**Output:** Estimated Story Point

**Begin**

1. Get ***user_story_text***

2. ***PreprocessText(user_story_text)*** → cleaned_text

3.  Convert text → lowercase

**4.**  ***Clean dataset*** → Remove duplicates, missing values

5.  ***Filter dataset*** → Keep only records where story_points ≤ 20

***6.  Rename columns***

7.   user_story → text

8.   estimated_story_points → story_points

**9.**  ***Split dataset*** → training_set = 80%, testing_set = 20%

10. ***BERTTokenizer(bert-base-uncased, max_length = 256)*** → tokenized_data

11.  Tokenizer output → {input_ids, attention_mask}

12.   Convert tokenized data into ***TensorFlow Dataset***

13.    Train = shuffle + batch_size = 8

14.     Test = batch_size = 8

*15.* ***Define Model Architecture***

16. Input: {input_ids, attention_mask}

17.  Pre-trained BERT Encoder → *(set to trainable = True)*

18.    LSTM layer *→ (64 units, Dropout = 0.3)*

19.    Intermediate Dense layer → *(32 units, Activation = ReLU)*

20.   Final Dropout layer (0.3) → *regularization*

21.  Output Dense Regressor → *(1 unit, Linear activation)*

22. **Compile Model**

23.  Optimizer → **Adam(lr = 2e-5, weight_decay = 0.01)**

24.   Loss → **MSE**

25.   Metric → **MAE**

***26. Training Procedure***

27. Apply Learning Rate Scheduler

28.  Execute training for maximum 10 epochs

*29.*   Apply Early Stopping → *(Monitor val_loss, patience = 3 epochs)*

30.   Restore best weights from the optimal epoch.

**31. Prediction and Mapping**

*32.*  Generate continuous prediction value $\hat{y}\;$ *→ unseen text.*

33.   Map $\hat{y}$ to the nearest value in agile Fibonacci set → *{1,2,3,5,8,13,20}*

34. Return → *Estimated_Story_Point*

**End**

#### 3.2.3. System Development for Effort Estimation.

**Fig 6:**
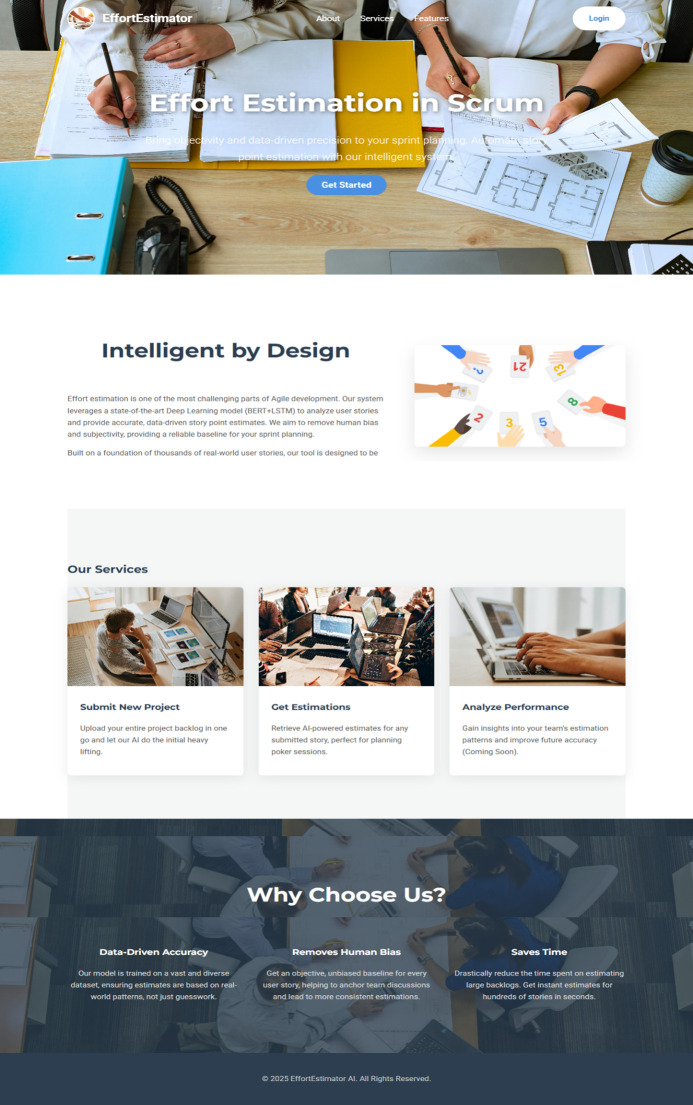
Web-based System developed for Effort Estimation.

**Fig 7:**
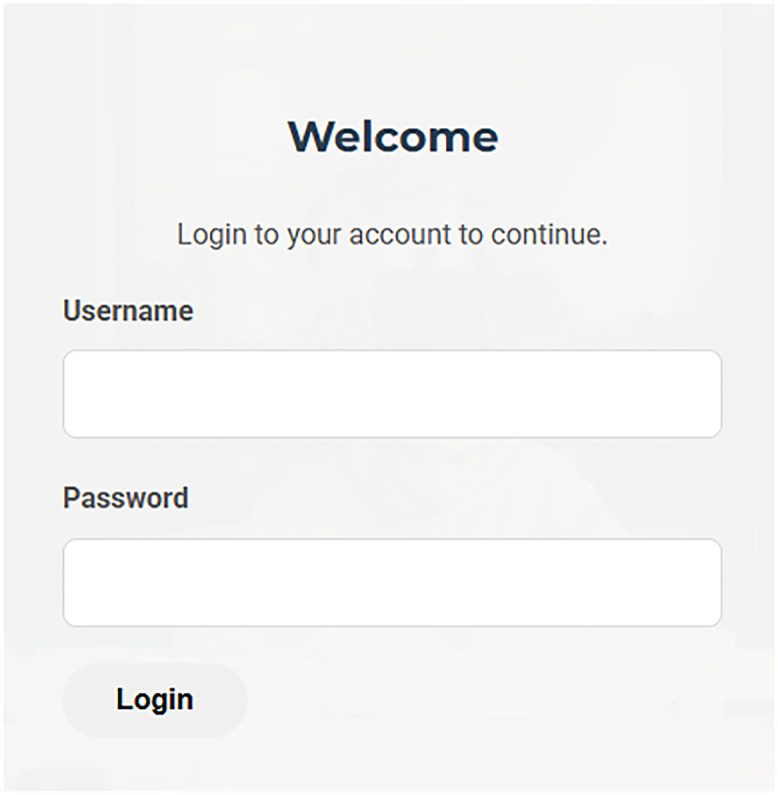
Login interface for the web-based estimation tool.

**Fig 8:**
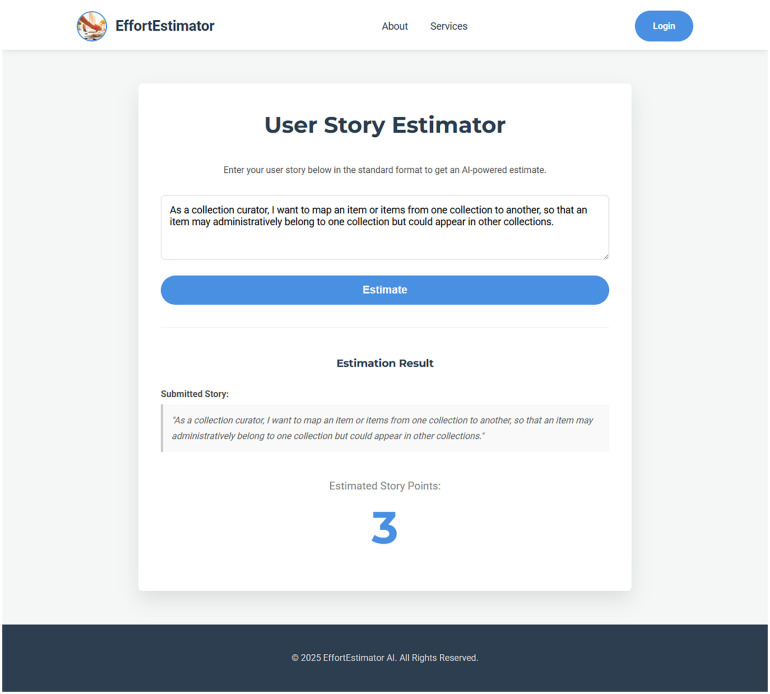
Estimation of single user story.

In order to bridge the gap between theoretical research and industry use, the proposed BERT-LSTM model was operationalized into a functioning web-based system. The system architecture is designed to provide ease to the agile practitioners, specifically scrum masters, in managing project backlogs effectively. The interface once lets for the submission of user stories in batches, from where users are able to select individual user stories to receive immediate AI-driven effort estimates. Upon prediction call, natural language text of user story is processed by backend algorithm in real-time. A critical feature of this system is the post-processing layer of mapping the raw and continuous numerical output of the model for the closest value on the standard Fibonacci sequence (1, 2, 3, 5, 8, 13, 20). This mapping helps ensure that the system offers useful practical estimates which follow industry-standard Planning Poker practices and thus they are directly usable for sprint planning. Furthermore, the developed system was evaluated from the same experts of the industrial scrum experts who assisted during the data collection phase where they tested the system using real-world data that verified the system to function correctly and provide reliable estimation support. The overall structure of the system design is shown in Fig 6, where the input interface and the final estimation output are given in Fig 7 and 8, respectively.

### 3.3 Prototype demonstration and evaluation

This phase consists of assessing the performance of the developed artifact and interpreting the results in order to answer the research questions.

#### 3.3.1. Evaluation of proposed model.

The objective of the evaluation phase was to evaluate the predictive accuracy of the proposed BERT-LSTM model rigorously as well as to assess the stability and generalization capability using a multiple metrics statistical model. To provide an objective evaluation, the curated dataset containing 4,079 unique user stories was divided into a 80/20 train-test split with 3,263 user stories representing 80% of the data used to train the model and the remaining 816 user stories constituting 20% as an independent testing dataset for model performance at index time by excluding them from training.

Performance was mainly measured in terms of MAE as formulated in Equation 1, which is a direct and linear measure of prediction error regarding story points and hence can be interpreted easily for agile practitioners [24]. To complement MAE and to evaluate how sensitive the model is to huge deviation, RMSE was computationally calculated, as shown in Equation 2, these values give much attention to outliers and therefore provide information about how reliable the architecture is for predicting high complexity requirements. Furthermore, the $R^{\text{2}}$ as described in Equation 3 was used along with an evaluation of the “Goodness-of-Fit” and the ability to fit the model to capture such variance in the effort values based on the textual semantic features. In addition to the proposed hybrid model, two baseline configurations (Standalone BERT and Standalone LSTM) were implemented to evaluate the individual contribution of each component. The mathematical formulations for these metrics are given below in the following:


$$\begin{array}{c} MAE\;=\frac{\text{1}}{n}\sum_{i=\text{1}}^{n}\;|y_{i}-\;{\hat{y}}_{i}| \end{array}$$ (1)

$$\begin{array}{c} RMSE\;=\;\sqrt{\frac{\text{1}}{n}\;\sum_{i=\text{1}}^{n}{\left|y_{i}-\;{\hat{y}}_{i}\right|}^{\text{2}}} \end{array}$$ (2)

$$\begin{array}{c} R^{\text{2}}\;=\;\text{1}\;-\;\left[\frac{{\sum_{i=\text{1}}^{n}\left|{\hat{y}}_{i}-\;y_{i}\right|}^{\text{2}}}{\sum_{i=\text{1}}^{n}{\left|y_{i}-\;{\bar{y}}_{i}\right|}^{\text{2}}}\right] \end{array}$$ (3)

Where n represents the number of samples, $y_{i}$ is the actual expert estimate, ${\hat{y}}_{i}$ is the model prediction and ${\bar{y}}_{i}$ is the mean of the actual expert estimates. Finally, to move beyond descriptive statistics and ensure the scientific validity of the performance gains, the results were subjected to a paired Wilcoxon Signed-Rank Test to confirm statistical significance (p<0.05) against all baseline comparisons.

## 4. Results

**Fig 9:**
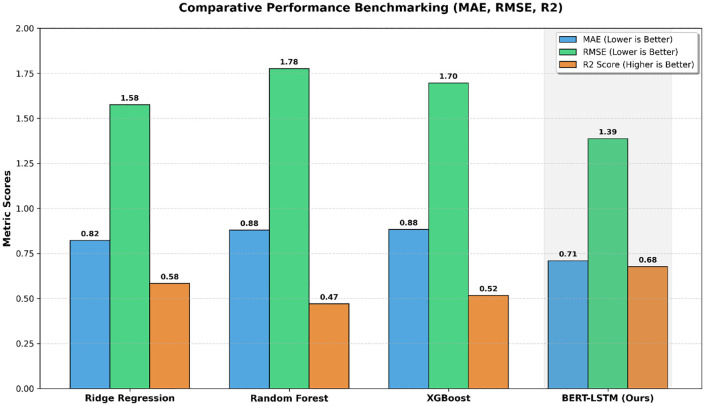
Comparative performance benchmarking of the proposed BERT-LSTM model against traditional ML baselines across MAE, RMSE, and $R^{\text{2}}$.

A comprehensive benchmarking study was performed on the expert-curated dataset to test the predictive reliability of the proposed hybrid model against existing baselines. The quantitative results, summarized in Table 4, and the model performance as a comparative model visualized in Fig 9, give an objective look at the performance of the models. The empirical evidence shows that the BERT-LSTM model has a huge improvement over traditional frequency based and ensemble-based ML algorithms. While ridge regression emerged as the strongest ML baseline having an MAE of 0.8640, the proposed hybrid DL model got a final MAE of 0.6481 which is a large 25.0% reduction in error. This improvement is also supported by the $R^{\text{2}}\;$ score of 0.6581, which is significantly greater than the baseline models which struggled in the range of 0.47 to 0.59. Such a divergence indicates the leveraging of transformer-based contextual embeddings, the DL model manages to understand deep semantics in user stories that the normal model using TF-IDF fail fundamentally to comprehend.

**Table 4:** Performance Comparison of the Proposed Model against ML Baselines.

Model	MAE	RMSE	R-squared ${(R}^{2}$)
Ridge Regression	0.8640	1.6028	0.5856
Random Forest	0.8868	1.7629	0.4795
XGBoost	0.9212	1.7964	0.4795
Proposed BERT-LSTM	0.6481	1.4559	0.6581

**Fig 10:**
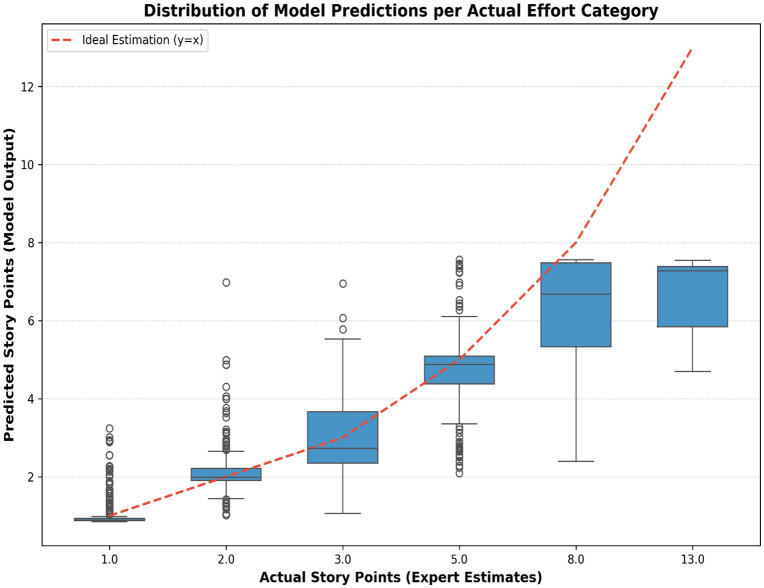
Distribution of model predictions across distinct story point categories relative to the ideal estimation baseline (𝐲=𝐱).

A granular analysis of the model’s predictions for different story point categories is shown in Fig 10. The distribution shows that the BERT-LSTM model achieves exceptional precision for the smaller user stories, namely in the story points 1–5, for which the predicted medians are close to expert estimates. However, for higher complexity stories (8 and 13) a conservative estimation trend is noticed, where the model tends to be a little less accurate than the effort should be. From a practical Scrum perspective this finding has a critical strategic importance, since the DL model has its largest reliability for low magnitudes of effort, the DL model is a perfect tool to help estimate the size of atomic requirements. As a result, it is recommended to follow a “Decomposition-First” strategy; whenever a high-complexity requirement or “Epic” is faced, it should be broken down into smaller and granular user stories.

**Fig 11:**
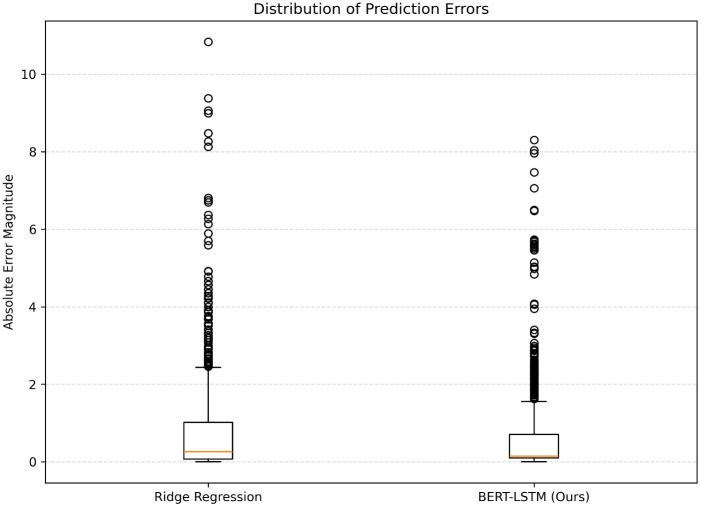
Comparative analysis of the absolute error distribution for the best-performing baseline and the proposed hybrid model.

By estimating these smaller units, practitioners may take advantage of the greater level of precision afforded by the model, so that a more accurate and data-driven Sprint Planning process may be obtained. The robustness and stability of the proposed model is further verified from the absolute error distribution analysis as shown in fig 11. When compared to the best performing ML baseline [Ridge Regression], the BERT-LSTM model is much closer in terms of the error spread and one of the lower median errors magnitude. While the ML baseline has a larger interquartile range, as well as more extreme outliers, the range of errors of the hybrid DL model is stable and small. This consistency shows that the integration through LSTM of sequential memory with semantic encoders such as BERT is a more robust estimation model. In order to further prove the stability of proposed model and deal with the possibility of partitioning bias, a supplementary 5-fold cross validation was conducted. The model achieved a MAE of 0.652 with a standard deviation of 0.018 across the five folds demonstrating its excellent stability and generalizability. These results are very similar to our main 80/20 split result of 0.65, which confirms that our results are not artifacts of a particular data partition. Ultimately, the results show that using this hybrid model is an effective way of reducing the subjectivity and volatility that surrounds manual agile estimation, achieving a new performance benchmark for automated effort prediction. The contribution of each component of the hybrid model is further analyzed through an ablation study presented in Section 4.2.

In order to match our regression-based effort estimates with the discrete and industry standard Fibonacci scale {1,2,3,5,8,13,20} used in Agile Scrum environments, a post hoc classification evaluation was performed. This transformation bridges the gap between the continuous output of DL models and the practical requirements of Sprint planning where practitioners requires categorized effort labels. As shown in Table 5, the classification performance shows an overall accuracy of 72%. Further, the model achieved a high score of 0.93 for the ‘1’ point category, highlighting the effectiveness of the model in capturing semantic patterns in the most common and low complexity user stories, thereby providing high predictive precision. While the precision score is expected to vary in higher effort classes, the average weighted F1 scores of 0.72 demonstrates the stability of the hybrid BERT-LSTM model in various tasks granularities.

**Table 5:** Classification Metrics per Story Point Category.

Story Points	Precision	Recall	F1-Score
1	0.98	0.88	0.93
2	0.78	0.80	0.79
3	0.41	0.57	0.48
5	0.58	0.62	0.60
8	0.67	0.47	0.55
13	0.00	0.00	0.00
20	0.00	0.00	0.00
**Accuracy**			**0.72**

**Fig 12:**
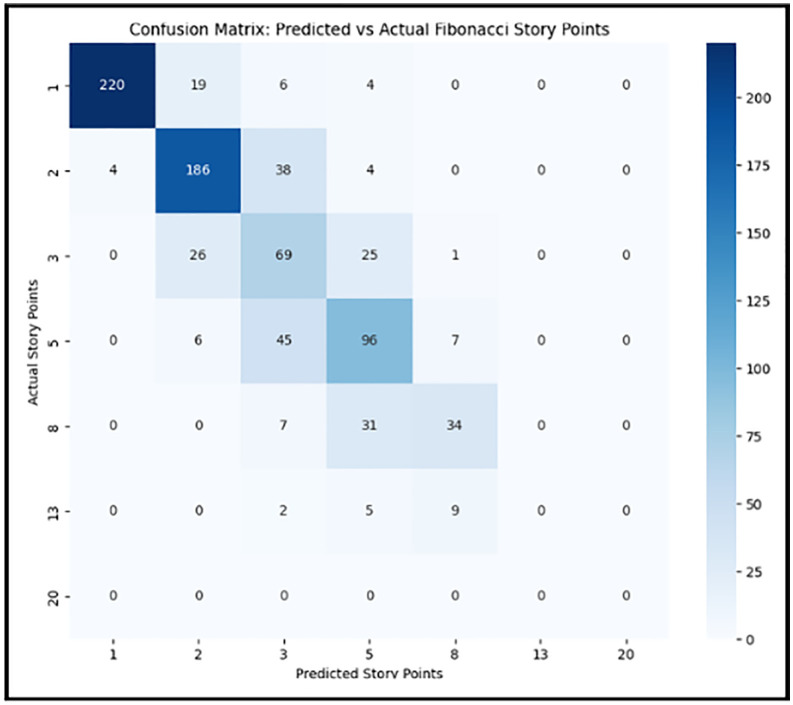
Confusion Matrix illustrating the classification performance of the BERT-LSTM hybrid model across standard Fibonacci story point categories.

The Confusion Matrix shown in Fig. 12 provides additional useful insights into the model’s predictive error distribution. A diagonal dominance is clearly observed in the matrix, indicating that most predictions are exactly in line with the ground-truth estimates provided by the experts. Importantly, the error distribution exhibits ‘neighbor-class’ misclassifications for numbers the errors are typically localized within the adjacent Fibonacci class numbers, e.g., a ‘2’ point requirement being estimated as ‘3’. In software effort estimation, these minor deviations are not considered statistically or practically acceptable since they are only marginal fluctuations and not a catastrophic failure in the estimation of the software effort. Moreover, the conservative estimating bias of the model towards higher effort categories (13 and 20 points) has a strategic function in the agile management. This under-estimation for large and complex requirements serves as an automatic complexity trigger, which makes ‘Decomposition-First’ the implicit default approach. The model helps to reduce risk of Epics by marking volatile requirements as targets for further refinement. Overall, the results indicate that the proposed architecture is able to provide superior regression precision and it can be used as a standardized, context-aware decision-support tool in agile project planning.

### 4.1. Statistical Significance Analysis

To determine whether the performance improvements of the proposed BERT-LSTM model against the baseline algorithms are statistically significant, a paired Wilcoxon Signed-Rank Test was performed. This non-parametric test was used due to the fact that prediction error distributions usually do not follow normality in agile effort estimation based tasks, which in turn makes non-parametric statistical tests more suitable. The test was performed on an absolute prediction errors produced by the proposed model and each baseline model (Linear Regression, Random Forest, and XGBoost) on the same testing dataset. For each user story in the test set, the absolute prediction error output by the BERT-LSTM model was compared with an error output from each baseline algorithm. The null hypothesis (H₀) assumes there is no significant difference between the prediction errors of the proposed model of the baseline models. The alternative hypothesis (H1) is that the proposed BERT-LSTM model works significantly better with a lower prediction errors. A significance level of alpha = 0.05 was used for significance. The results of this statistical analysis, which are presented in Table 6, show that the proposed model has better statistical results compared to all the baseline algorithms, at a 95% confidence level (p < 0.05). The BERT – LSTM model shows significant improvements over Linear Regression (p < 0.00001), Random Forest (p < 0.00001) and XGBoost (p < 0.00001). Since all of the calculated p-values are less than the significance value, the null hypothesis is rejected for all comparisons.

**Table 6:** Statistical Significance Results of the Proposed BERT-LSTM Model against ML Baselines.

Baseline Model	Wilcoxon p-value	Statistical Significance
Bert-LSTM vs Ridge Regression	p < 0.001	Yes
Bert-LSTM vs Random Forest	p < 0.001	Yes
Bert-LSTM vs XGBoost	p < 0.001	Yes

These results give statistical evidence that what they found with the improvements in prediction accuracy is unlikely to be chance in the dataset. Instead, the results indicate that the proposed hybrid model is more successful for extracting semantic and contextual information from stories in user stories than traditional ML approaches.

To further investigate the contribution of the proposed hybrid model, an ablation study was conducted comparing the hybrid model with standalone BERT and standalone LSTM configurations.

### 4.2. Ablation Study

To systematically examine and justify the architectural additions of the proposed hybrid model, an ablation study was performed, and the findings are summarized in Table 7. The main goal of this analysis is to make a clear comparison between the proposed hybrid architecture and its base components (BERT-only and LSTM-only). This comparison determines whether the superior predictive performance is a direct consequence of hybrid synergy or is a result of characteristics of the datasets. To this end, two simplified, standalone configurations were setup and evaluated with the same set of data.

**Table 7:** Ablation Study: Impact of Hybrid Model.

Model Configuration	MAE	RMSE	$R^{2}$
Standalone BERT (Contextual only)	0.8134	1.8215	0.4912
Standalone LSTM (Sequential only)	0.9245	2.0541	0.4134
Proposed Hybrid BERT-LSTM	0.6481	1.4559	0.6581

The empirical results presented in Table 7 vividly illustrate that the individual base models fail to achieve the predictive accuracy of the proposed hybrid model. The Standalone BERT model (MAE: 0.8134) was able to capture deep semantic and contextual representations from the agile requirements in isolation, but did not have an explicit structural mechanism that models the sequential and temporal narratives (like “As a… I want… so that…”) in a user story. The Standalone LSTM model (MAE: 0.9245), on the other hand, was able to manage these sequential structures very well, but prediction error was much higher due to the lack of rich and useful pre-trained context embeddings. The impressive improvement is also clearly shown by explicitly comparing these standalone components with the proposed Hybrid BERT-LSTM model (MAE: 0.6481) and it is not just an incremental increase. The hybrid architecture is a good solution to the shortcomings of the base elements. It combines the powerful long-range sequential dependency modeling capability of the LSTM with the bidirectional contextual understanding of BERT. The results show, for the first time, that this fusion is indeed required to comprehensively capture the linguistic complexity of unstructured agile narratives, justifying the proposed architecture.

### 4.3. Comparison with state of art

The benchmarking results summarized in Table 8 give empirical evidence of the superiority of the proposed hybrid BERT-LSTM model with respect to semantic depth, data integrity, and statistical robustness. While pioneering works such as Choetkiertikul et al. [23] were able to introduce LSTMs for the problem of effort estimation, the use of older Word2Vec embedding and the lack of consideration of statistical significance make the scientific validity of those findings limited. Similarly, although Alsaadi & Saeedi [10] achieved a respectable MAE of 0.78, their results were purely descriptive without any statistical proof of significance, and were further calibrated on a microscopic dataset of students.

**Table 8:** Comparative Analysis with State-of-the-Art Literature.

Author(s)	Methodology	Dataset	Results	Key Contribution & Technical Limitations
Choetkiertikul et al. [23]	LSTM + Features	JIRA (~23k Stories)	MAE = 0.81–1.45	Introduced LSTMs for story point estimation, but relied on static Word2Vec embeddings. No statistical significance testing was reported to validate model superiority.
Alsaadi & Saeedi [10]	Ensemble ML	Students (140 Stories)	MAE = 0.78	Demonstrated the efficacy of ensemble learning, but constrained by a tiny academic dataset. Performance gains were not mathematically validated via significance tests.
Kumar & Vidyulatha [15]	ELM	Desharnais (Structured)	(90% + Acc)	Fast and efficient ELM model, but ignores textual narratives by relying on metadata. Lacks rigorous statistical comparison with alternative architectures.
Tawosi et al. [18]	Deep-SE (Replication)	JIRA (~31k Stories)	MAE > 2.00	Massive-scale replication study revealing model failure on uncurated data. Theoretical comparison was provided without paired statistical significance analysis.
Rasheed et al. [17]	Ensemble (RR + ET + MLP)	Real-world (159)	MAE = 0.68	Robustness validation via cross-dataset benchmarking but lacks deep semantic extraction.
Proposed Model	Hybrid BERT-LSTM	Expert-Curated (4,079)	MAE = 0.6481	Directly overcomes data-quality and semantic gaps via BERT and expert-curated data; achieves the lowest MAE, validated as statistically significant via Wilcoxon test (p < 0.05)

Unlike the metadata-driven approaches such as the ELM model by Kumar & Vidyulatha [15], which do not work with the textual stories and lack a statistically sound performance leap, the present study infers complexity directly from the text. Furthermore, the massive-scale replication study by Tawosi et al. [18] showed the unreliability of DL on noisy data; this research overcomes that limitation by combining high-quality curation with a stable BERT-LSTM architecture. While recent work by Rasheed et al. [17] introduced an ensemble of Ridge Regression, Extra Tree and MLP achieving an MAE of 0.68, the proposed BERT-LSTM model resolves these gaps by using the BERT Transformer encoder and performing a rigorous Wilcoxon Signed-Rank Test.

This study results in a far superior score of 0.6481 MAE that is mathematically proven to be superior to existing benchmarks (p < 0.05). Furthermore, this work addresses the gap between the precision of the models and industrial generalizability, which is one of the biggest bottlenecks in prior academic attempts. A major differentiator of this research corresponds to the shift from purely theoretical comparisons to experimentally validated ones, delivering the first performance benchmark to derive deep semantic understanding with a stable RMSE of 1.4559 and $R^{\text{2}}$ of 0.6581. Ultimately, by combining high-quality curation with a stable BERT-LSTM architecture, this study enables the highly reliable use of decision support to estimate agile effort.

### 4.4. Answer to Research Question

The findings of this study give a precise answer to the research question by showing that a hybrid DL model could be effective in improving the accuracy of the user story effort estimation. As explained in Section 3.2.2 the proposed model combines the BERT-based contextual embeddings and LSTM layer to naturally capture both semantic and sequential patterns that are present in user story narrative. Using the expert-curated dataset discussed in Section 3.2.1, the model achieved a MAE 0.6481 and an $R^{\text{2}}$ score of 0.6581 which is a 25% reduction in prediction error compared to other traditional ML baselines such as Ridge Regression detailed in Section 4. Furthermore, Wilcoxon Signed-Rank Test was used to confirm that the observed improvements are statistically significant (p < 0.05), which indicates that the proposed model can provide a reliable and objective alternative to manual estimation. Although the model shows the highest accuracy for smaller user stories (1–5 Story Points), results suggest that the integration of the proposed model in Scrum workflows when particularly large requirements are split into smaller stories can significantly reduce the subjectivity and variability inherent in human-led effort estimation.

## 5. Conclusion and future work

This study addresses the challenge of accurate user story effort estimation in agile software development by introducing a hybrid DL model which combines semantic understanding with sequential modeling. Combining the pre-trained BERT encoder with an LSTM network, the model can effectively learn contextual and structural patterns in the user story narrations, which can be used to produce more accurate and data-driven predictions. One of the important contributions of this study is that it has created a large scale dataset of 6,956 user stories that have been comprehensively preprocessed and filtered to 4,079 high quality stories through expert validation. The curated dataset offers a solid base to enhance the accuracy of DL based estimation models. The proposed model demonstrates superior performance, achieving a MAE of 0.6481, RMSE of 1.4559, and a Coefficient of Determination $R^{\text{2}}$ of 0.6581. Furthermore, when mapping the continuous regression outputs to the discrete Fibonacci sequence used in Scrum, the model achieves a classification accuracy of 72%, demonstrating its direct applicability to practical sprint planning. The findings support the efficacy of the hybrid model in improving predictive accuracy in effort estimation. Moreover, the statistical validation with the Wilcoxon Signed Rank Test (p < 0.05) confirms the fact that the improvements are statistically significant and not a mere coincidence. Besides its methodological contributions, the model is also deployed as a web-based decision support system, which proves its practical applicability in estimating real-time in agile environments. Altogether, the research identifies the possibility of using large scale curated datasets and hybrid DL methods to enhance the objectivity of the subject and the reliability of estimations in Scrum-based project management. Future efforts will concentrate on incorporating XAI methods and testing the model on real world industrial data to promote transparency and generalizability.

## References

[1] Edison H, Wang X, Conboy K. Comparing Methods for Large-Scale Agile Software Development: A Systematic Literature Review. IIEEE Trans Software Eng. 2022;48(8):2709–31. doi: 10.1109/tse.2021.3069039

[2] Pasuksmit J, Thongtanunam P, Karunasekera S. A systematic literature review on reasons and approaches for accurate effort estimations in agile. ACM Computing Surveys. 2024;56(11):1–37.

[3] Iqbal M, Ijaz M, Mazhar T, Shahzad T, Abbas Q, Ghadi Y, et al. Exploring issues of story-based effort estimation in Agile Software Development (ASD). Science of Computer Programming. 2024;236:103114. doi: 10.1016/j.scico.2024.103114

[4] Poženel M, Fürst L, Vavpotič D, Hovelja T. Agile Effort Estimation: Comparing the Accuracy and Efficiency of Planning Poker, Bucket System, and Affinity Estimation Methods. arXiv preprint. 2024. doi: arXiv:2401.16152

[5] Hamid M, Zeshan F, Ahmad A, Malik S, Saleem M, Tabassum N, et al. Analysis of Software Success Through Structural Equation Modeling. Intelligent Automation & Soft Computing. 2022;31(3):1689–701. doi: 10.32604/iasc.2022.020898

[6] Hamid M, Zeshan F, Ahmad A. Fuzzy logic-based expert system for effort estimation in scrum projects. In: 2021 International Conference on Decision Aid Sciences and Application (DASA). Sakheer, Bahrain. 2021. 761–5. doi: 10.1109/DASA53625.2021.9682343

[7] Yang Y, Xia X, Lo D, Grundy J. A survey on deep learning for software engineering. ACM Computing Surveys. 2022;54(10s):1–73.

[8] Vyas M, Bohra A, Lamba CS, Vyas A. A Review on Software Cost and Effort Estimation Techniques for Agile Development Process. International Journal of Recent Research Aspects. 2018;5(1):1–5.

[9] Mahmood Y, Kama N, Azmi A, Khan AS, Ali M. Software effort estimation accuracy prediction of machine learning techniques: A systematic performance evaluation. Softw Pract Exp. 2021;52(1):39–65. doi: 10.1002/spe.3009

[10] Alsaadi B, Saeedi K. Ensemble effort estimation for novice agile teams. Information and Software Technology. 2024;170:107447. doi: 10.1016/j.infsof.2024.107447

[11] Hariyanti E, et al. The implementation of machine learning for software effort estimation: A literature review. Khazanah Informatika: Jurnal Ilmu Komputer dan Informatika. 2024;10(1):47–57.

[12] Lavazza L, Locoro A, Meli R. Using Machine Learning and Simplified Functional Measures to Estimate Software Development Effort. IEEE Access. 2024;12:142505–23. doi: 10.1109/access.2024.3471428

[13] Arora M, Verma S, Wozniak K, Shafi J, Ijaz MF. An efficient ANFIS-EEBAT approach to estimate effort of Scrum projects. Scientific Reports. 2022;12(1):7974. 35562362 10.1038/s41598-022-11565-2 PMC9106679

[14] Turic M, Celar S, Miletic T. Advanced Bayesian Network for Task Effort Estimation in Agile Software Development: A Case Study. In: 2023 46th International Convention on Information, Communication and Electronic Technology (MIPRO), 2023. 1391–6. 10.23919/MIPRO57284.2023.10159987

[15] Hamid M, Zeshan F, Ahmad A, Ahmad F, Hamza MA, Khan ZA, et al. An Intelligent Recommender and Decision Support System (IRDSS) for Effective Management of Software Projects. IEEE Access. 2020;8:140752–66. doi: 10.1109/access.2020.3010968

[16] Kumar RA, Vidyulatha G. Extreme learning machine applied to software development effort estimation. African J Biological Sciences. 2024;6(7):746–57.

[17] Rasheed M, Fatima I, Fatima D, Hamid M, Hajjej F. Effort estimation in scrum using AI. Autom Softw Eng. 2026;33(2). doi: 10.1007/s10515-026-00607-y

[18] Tawosi V, Moussa R, Sarro F. Deep learning for agile effort estimation: have we solved the problem yet?. In: arXiv preprint, 2024. https://doi.org/arXiv:2403.01358

[19] Fatima I, Rasheed M, Fatima D, Hamid M, Jaghdam IH, Zahary AT. A novel stacking-based ensemble machine learning model for accurate user story effort estimation in scrum. Journal of Big Data. Available at: https://github.com/drmhamid/Effort_estimation_of_user_stories_in_scrum_model. 2026.

[20] Fatima I, Rasheed M, Fatima D, Hamid M, Jaghdam IH, Zahary AT. A novel stacking-based ensemble machine learning model for accurate user story effort estimation in scrum. J Big Data. 2026;13(1). doi: 10.1186/s40537-026-01414-8

[21] https://github.com/hash-7201/UserStories-Dataset

[22] Appcelerator. The Appcelerator repository. https://jira.appcelerator.org. 2016.

[23] Choetkiertikul M, Dam HK, Tran T, Pham T, Ghose A, Menzies T. A Deep Learning Model for Estimating Story Points. IIEEE Trans Software Eng. 2019;45(7):637–56. doi: 10.1109/tse.2018.2792473

[24] Hodson TO. “ Root-Mean-Square Error (RMSE) or Mean Absolute Error (MAE): When to Use Them or Not,” Geoscientific Model Development, vol. 15, pp. 5481–7, 2022, doi: 10.5194/gmd-15-5481-2022

